# The Importance of Eculizumab in the Treatment of Atypical Hemolytic Uremic Syndrome

**DOI:** 10.7759/cureus.25743

**Published:** 2022-06-08

**Authors:** Mariana S Santos, Sofia Ventura, Abel Alves, Raquel Cabral, Manuela Henriques

**Affiliations:** 1 Department of Intensive Care Medicine, Hospital do Divino Espírito Santo de Ponta Delgada, Ponta Delgada, PRT; 2 Department of Nephrology, Hospital do Divino Espírito Santo de Ponta Delgada, Ponta Delgada, PRT

**Keywords:** plasmapheresis, prognosis, eculizumab, mortality, ahus

## Abstract

In most cases, the atypical hemolytic uremic syndrome (aHUS) is a genetic disease resulting from defects in the regulation of the complement cascade, which conditions the development of thrombotic microangiopathy. Clinically, aHUS presents with acute kidney injury, thrombocytopenia, and microangiopathic hemolytic anemia, with frequent renal and extrarenal thrombotic phenomena. Early diagnosis and treatment are essential. Plasmapheresis is an alternative treatment being frequently used considering the high cost of eculizumab, despite its lower clinical efficacy. This article describes the clinical case of a patient admitted to intensive care with a personal history of aHUS diagnosed 10 years ago, with recurrent aHUS triggered by viral infection. The patient presented with acute kidney injury and thrombocytopenia. Despite the institution of admission of plasmapheresis, the clinical evolution was only favorable after the administration of eculizumab, highlighting the importance of early initiation of this therapy.

## Introduction

Hemolytic Uremic Syndrome (HUS) is characterized by acute kidney injury, thrombocytopenia, and microangiopathic hemolytic anemia [1,2]. Two types of HUS are distinguished: typical HUS (most common, about 90%) [3], caused by Shiga toxin, which is produced by the *Escherichia coli* serotype O157:H7 [4], diagnosed through the identification of the bacteria in coproculture, the toxin by immunoassays, polymerase chain reaction (PCR), and/or serum antibody screening.

Atypical HUS (aHUS) is rarer and is mainly a genetic disease that results from defects in the regulation of the complement cascade. It constitutes hyperactivity of the alternative complement pathway, originating from a thrombotic microangiopathy process [1]. The aHUS can occur at any age, and it can be sporadic or familial. Diagnosis is difficult, excluding other causes of thrombotic microangiopathy. The prognosis of this disease is poor, with high mortality and morbidity in an acute phase, with a high risk of renal and extrarenal thrombotic events. The most common extrarenal manifestations are hematologic manifestations that include absolute or relative thrombocytopenia and microangiopathic hemolytic anemia and renal ischemia (50% of which can result in end-stage chronic kidney disease) [2], and cerebral ischemia [5,6]. The least common are manifestations of the gastrointestinal tract and heart [5,6].

Eculizumab is a monoclonal antibody with an affinity for the C5 complement protein. This blocks the formation of C5a and the C5b-9 complex, inhibiting this complement and decreasing thrombotic microangiopathic activity [6]. Eculizumab can be used as a specific therapy for HUS. However, it is not easily accessible due to its high cost and the extensive bureaucratic authorization process associated with its use. Thus, the alternative treatment, plasmapheresis, is the most used one [6,7].

The authors report the case of a patient with aHUS subjected to plasmapheresis as initial treatment, but showing clinical improvement only after beginning treatment with eculizumab.

## Case presentation

We describe the case of a male patient, 20 years old, with a history of aHUS diagnosed 10 years ago, with a deficit of complement (C3 and CH50) and arterial hypertension medicated with enalapril. He appeared in the emergency department due to fever, odynophagia, and headache that had evolved over three days.

During an objective examination, he was conscious and oriented, eupneic and normotensive; jaundiced skin and mucous membranes, highlighting non-pruritic erythematous papules affecting the face, feet, hands, and abdominal wall. Analytical test results revealed thrombocytopenia, low reticulocytes, non-doseable lactate dehydrogenase (LDH), unchanged coagulation, acute kidney injury, a cytocholestasis pattern, and total hyperbilirubinemia with high direct bilirubin (Table 1). The abdominal ultrasound was without changes.

**Table 1:** First analysis at the Emergency Service. INR: international normalized ratio.

Laboratory investigations	Patient lab	Reference range
Hemoglobin	14.0>12.2	14-18 g/dL
Leukocytes	8.58	4-11.5×10^3^/µl
Platelets	30	150-400×10^3^/µl
Reticulocytes	0.7	0.5–2.5%
Prothrombin Time	13.1	9.4–13.4 s
INR	1.14	0.75–1.22
Activated Partial Thromboplastin Time	33.5	23–37.0 s
Urea	Hemolyzed sample	10–50 mg/dL
Creatinine	1.73	0.7–1.3 mg/dL
Aspartate aminotransferase	Hemolyzed sample	5–34 U/L
Alanine Aminotransferase	285	0–55 U/L
Alkaline phosphatase	111	46–115 U/L
Gamma Glutamyltransferase	266	<73 U/L
Lactic Dehydrogenase	Hemolyzed sample	125–220 U/L
Total bilirubin	9.03	0.3–1.2 mg/dL
Indirect bilirubin	3.55	<0.31 mg/dL
C-reactive protein	7.86	0–0.5 mg/dL

A peripheral blood smear showed several schizocytes. From the additional complementary study, the serum detection of Enteroviruses by PCR is highlighted, and therefore relapse of aHUS probably precipitated by coxsackievirus infection (hand-foot-mouth syndrome) was assumed. Viral serologies were also carried out for Epstein-Barr virus (EBV), cytomegalovirus (CMV), parvovirus, human immunodeficiency virus (HIV), hepatitis C virus (HCV), hepatitis B virus (HBV), and syphilis and PCR for influenza, herpes simplex virus (HSV), and adenovirus that proved negative, as well as cultural examinations of conventional microbiology, namely the search for film-arrays of *Escherichia coli.*

**Figure 1:**
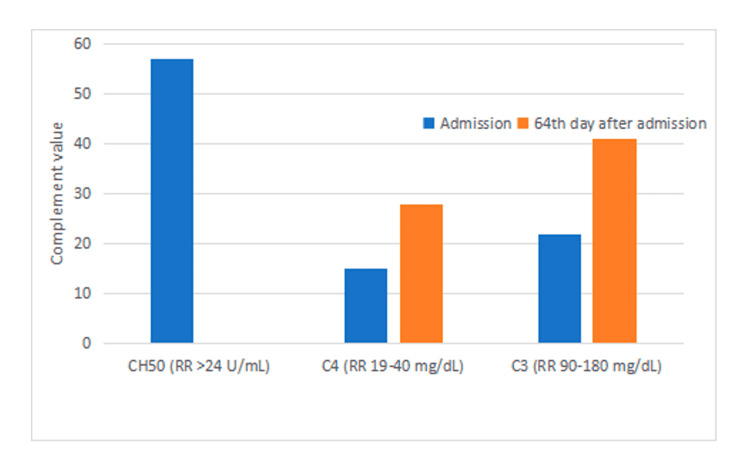
Assaying of C3, C4 and CH50 on admission and consultation. RR: Reference range.

He was admitted to the Intensive Care Unit. Considering the history of aHUS, associated recurrence was considered likely, and due to the immediate unavailability of eculizumab in the hospital, plasmapheresis was started as an alternative treatment. Complement C3 assay was high, and C4 was normal (Figure 1).

**Figure 2:**
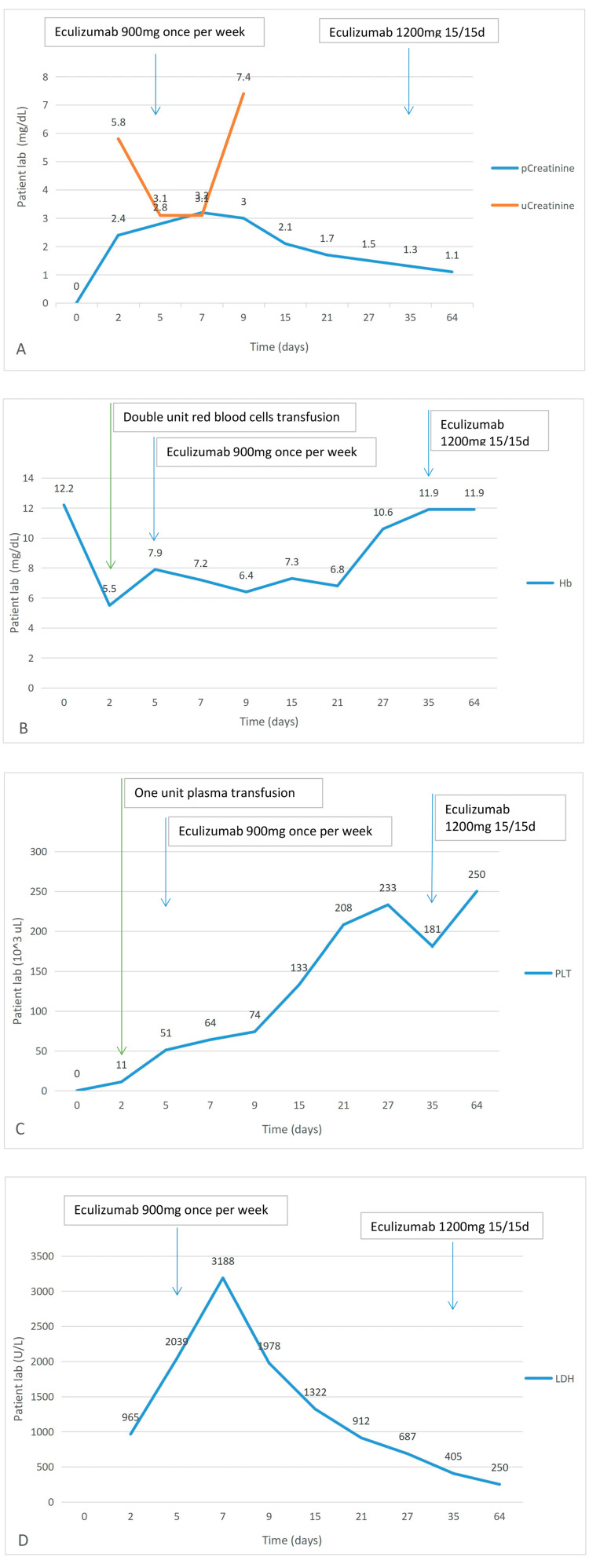
Evolution of analytical parameters in inpatient/outpatient. (A) Evolution of plasma creatinine and creatinuria. (B) Evolution of hemoglobin. (C) Evolution of lactic dehydrogenase. (D) Evolution of Platelets. pCreatinine: plasma creatinine; uCreatinine: creatinuria; d: day; Hb: hemoglobin; PLT: platelets; LDH: lactic dehydrogenase.

One plasmapheresis session per day was started, and on the third day, there was a need for two sessions per day due to the worsening of severe hemolytic anemia, thrombocytopenia, and renal function. The patient underwent six plasmapheresis sessions in the first five days of hospitalization (with a replacement of 1.5 volume of plasma 60-75 mL/kg in each session). Despite the treatment instituted, hemolysis was maintained with anemia and thrombocytopenia (Figure 2), which motivated transfusion support. This has a little benefit, with the patient remaining hypertensive and requiring labetalol in continuous infusion.

Considering the lack of improvement, he was started on corticosteroid therapy with methylprednisolone 500 mg id (10 mg/kg/day) on the third day of hospitalization and eculizumab (900 mg once per week) on the fifth day of hospitalization for four weeks (he also started ceftriaxone for meningitis prophylaxis considering the absence of vaccination for type B meningococcus). He completed four doses of eculizumab with clinical and analytical improvement, with an increase in hemoglobin (Hb) and platelets, recovery from kidney injury, decreased hemolytic process with a decrease in LDH, and normalization of Bilirubin (Figure 2).

He was transferred to the nephrology ward after the 12th day and was discharged from the hospital on the 15th day of admission. He is still being followed up in the nephrology outpatient consultation, where stable hemoglobin and platelets and progressive improvement in renal function were verified (Figure 2). He remained medicated with 900 mg eculizumab weekly for one month and then fortnightly at a dose of 1200 mg, maintaining the improvement profile. According to the guidance of the immunoallergology department he was vaccinated for Meningitis B.

## Discussion

The availability of eculizumab revolutionized the prognosis of patients with severe forms of aHUS presentation. This monoclonal antibody has an affinity for the C5 complement protein and leads to its inhibition. It is recommended to start eculizumab in the first 24-48 h after diagnosis or acute recurrence of aHUS [6-8]. However, because it is expensive and requires an extensive bureaucratic process, it is not available in most hospitals.

This therapy is recommended [6] as the first-line treatment in severe forms of presentation, achieving a significant decrease in thrombotic microangiopathic activity [3,9,10]. Eculizumab should be given early to increase the survival rate of patients [8]. Despite several plasmapheresis sessions, there was significant clinical improvement only after starting eculizumab in the clinical case presented. Based on the literature, in most patients with recurrent aHUS after the first episode, mortality was greater than 25%, and 50% of patients need dialysis in the next 12 months [2,11], yet varying according to the complement affected [8,12]. C3 mutations usually develop severe disease, with 60-70% of patients progressing to end-stage chronic kidney disease (ESRD) within the first year after presentation [13]. Recurrent disease is common in patients after kidney transplantation [13-16].

In the case presented here, there was no need to start hemodialysis within 12 months, maintaining a fortnightly dose of eculizumab and follow-up in consultation with the nephrology and immunollergology departments. Comparing eculizumab with plasmapheresis, several studies have shown that there is a significant improvement in renal function and hematological parameters [10] and 80% of patients discontinue hemodialysis (HD) [17]. Some studies in which plasmapheresis was started as an isolated treatment revealed that only 50% of patients had partial improvement in renal and hematological function [8]. Some authors [4,7,11] thus suggest that eculizumab should be started early if plasmapheresis is not effective, if there are complications inherent to the technique, or if there are potentially fatal complications of aHUS.

## Conclusions

Overall, aHUS is a rare disease characterized by acute kidney injury, thrombocytopenia, and microangiopathic hemolytic anemia. In severe forms of aHUS, the early availability of eculizumab can significantly improve the prognosis of these patients. In this context, despite the high costs, we consider it important that hospitals implement protocols that allow for their rapid availability.
